# Accuracy of analysis of cfDNA for detection of single nucleotide variants and copy number variants in breast cancer

**DOI:** 10.1186/s12885-019-5698-x

**Published:** 2019-05-17

**Authors:** Xin Yang, Kuo Zhang, Caiji Zhang, Rongxue Peng, Chengming Sun

**Affiliations:** 1 Department of Clinical Lab, Yantai Yuhuangding Hospital, 20 Yudong Road, Yantai, Shandong Province 264000 People’s Republic of China; 2 National Center for Clinical Laboratories, Beijing Hospital, National Center of Gerontology, Beijing, 100730 People’s Republic of China

**Keywords:** *PIK3CA*, *TP53*, *ESR1*, cfDNA, Mutation, Breast cancer, Single nucleotide variation, Copy number variation

## Abstract

**Background:**

Gene variants are dependable and sensitive biomarkers for target-specific therapies in breast cancer (BC). However, detection of mutations within tissues has many limitations. Plasma circulating free DNA (cfDNA) has been reported in many studies as an alternative tool for detection of mutations. But the diagnostic accuracy of cfDNA for most mutations in BC needs to be reviewed. This study was designed to perform comparative assessment of the diagnostic performance of cfDNA and DNA extracted from tissues for detection of single nucleotide variants (SNV) and copy number variants (CNV).

**Methods:**

True-positive (TP), false-positive (FP), false-negative (FN), and true-negative (TN) values were extracted from each selected study. Pooled sensitivity, specificity, positive likelihood ratio (PLR), negative likelihood ratio (NLR), and diagnostic odds ratio (DOR) were calculated. Subgroup analysis and single study omitted analysis were performed to quantify and explain the study heterogeneity.

**Results:**

Twenty eligible studies that involved 1055 cases were included in this meta-analysis. SNV studies in early breast cancer (EBC) subgroup are not suitable for meta-analysis owing to high heterogeneity. However, in advanced breast cancer (ABC) subgroup, the pooled sensitivity and specificity of detection of SNVs were 0.78 (0.71–0.84) and 0.92 (0.87–0.95), respectively. The summary receiver operative curve (SROC) exhibited an area under the curve (AUC) of 0.91(0.88–0.93). The pooled results of studies involving subgroups of *PIK3CA*, *TP53,* and *ESR1* indicate that the diagnostic value of different genes is different, such as AUC for *PIK3CA* and *TP53* were reported to be 0.96 (0.94–0.98) and 0.94 (0.91–0.95), respectively, and *ESR1* had the lowest diagnostic value of 0.80 (0.76–0.83). Owing to the low sensitivity and AUC in the cases of CNV, there is no value for cfDNA-based detection of CNV based on insufficient amount of CNV data.

**Conclusion:**

This meta-analysis suggests that the detection of gene mutations in cfDNA have adequate diagnostic accuracy and can be used as an alternative to the tumor tissue for detection of SNV but not for CNV in BC yet.

## Background

Breast cancer (BC) is the most common malignant tumor and the leading cause of cancer-associated death in women worldwide [1]. Studies have shown that mutations in genes related to BC can be used as biomarkers and allow personalized therapy for BC patients [2].These genes include *PIK3CA, TP53, ESR1*, and *ERBB2* [3–12]. Sensitivity to specific drugs such as everolimus is determined by the somatic mutational status of *PIK3CA* [10, 13]. APR-246 (PRIMA-1 MET) can target mutant *TP53* [14, 15] and *ESR1* gene mutations govern the use of anti-estrogen drugs for breast cancer treatment. Single nucleotide variants (SNV) and copy number variants (CNV) are the most common types of mutation in these genes related to BC [5, 16–18].

Traditionally, the identification of somatic mutations associated with cancer relies on the sequencing of the DNA isolated from the biopsy specimens. However, there are many disadvantages in this method, since it is invasive and repeated biopsies often yield variable results owing to intra-tumor heterogeneity [19]. Recent studies have shown that the genomic mutations in solid malignant tumors can be identified using cell-free DNA (cfDNA) released from cancer cells into blood circulation. This method forms a noninvasive blood test named “liquid biopsy” [20]**.** The analysis of cfDNA for detection of mutations may play a major role in personalized cancer treatment owing to many advantages including: (i) a noninvasive method for the detection of clinically useful mutations to guide therapy selection [21]; (ii) early detection of mutations related to resistance to a targeted treatment [20, 22]; (iii) a sensitive method for tracking patient’s response to therapy [23]; (iv) minimization of the influences from tumor heterogeneity.

A large number of studies confirm that cfDNA can be used as an alternative tool for the identification of BC biomarkers that provides the ability to overcome the drawbacks of invasive tissue biopsies but the results of these studies are variable. A systematic review and meta-analysis has been published for the analysis of cfDNA based detection accuracy of *PIK3CA* mutations [24]. However, this study does not review the literature available for detection of mutations in other genes related to breast cancer. In this study, we will perform a systematic review and meta-analysis to integrate the findings of different studies involving the use of cfDNA for the identification of SNVs and CNVs in the most common genes related to BC to comprehensively evaluate the accuracy of cfDNA-based detection of gene mutations in BC.

## Method

### Literature research strategy

This meta-analysis was performed and reported according to the guidelines about the diagnostic studies [25, 26]. PubMed, EMBASE were searched to identify suitable studies up to the July 30, 2018 and no start data limit was applied. A systematic and comprehensive search was performed with the combination of search terms “ circulating tumor DNA ” or “ circulating tumor-specific DNA ” or “ circulating DNA ” or “ Cell-free DNA ” or “ free DNA ” or “ plasma DNA ”, and “ breast ” or “ breast carcinoma ” or “ tumor of breast ” or “ breast neoplasms ” or “ breast tumor ”. No language restriction was set for a more comprehensive analysis, but only English articles were included.

### Inclusion and exclusion criteria

Eligible studies were selected based on the following inclusion criteria: i) studies that involve the evaluation of the accuracy of detecting gene mutations in BC patients using cfDNA; ii) studies that include the verification of gene mutations identified with cfDNA following the analysis of tumor tissues; iii) the studies that carry enough data to construct a diagnostic 2 × 2 table; and iv) studies that include that data for more than five patients.

The exclusion criteria included: i) Lack of verification of gene mutations by the analysis of tumor tissues; ii) insufficient data for constructing the 2 × 2 table; iii) reviews, comments, retracted studies, studies in languages other than English and those not on humans; and iv) evaluation of samples from less than five patients.

All the records were reviewed by the two authors (XY and KZ) independently and the consensus was drawn from each eligible study.

### Data extraction

The data were independently extracted from the included studies by three authors (XY, KZ and RXP). The fourth author (CJZ) input the data and the fifth author (CMS) assessed the data as well as resolved any disagreements. The data extracted or calculated from the articles included the author’s name, publication year, age and pathological stage of the participants, detection methods for different kinds of samples, assay indicators and mutation type, true positive (TP), false positive (FP), false negative (FN), and true negative (TN). With various detection methods, those with best sensitivity or specificity were preferred. In some studies without the original data for TP, FP, TN, FN, the accordance, sensitivity and specificity of gene mutation detection in tissue and plasma were available. Then according to the total number of samples (n = TP + FP + TN + FN), sensitivity [= TP/ (TP + FN) × 100%], specificity [= TN/ (TN + FP) × 100%] and overall coincidence rate [= (TP + TN) / (TP + FP + TN + FN) × 100%], the original TP / FP / FN / TN data can be calculated.

### Quality assessment

Quality of methodology of the included studies was evaluated based on quality assessment of diagnostic accuracy studies-2 (QUADAS-2) [27]. QUADAS-2 encompasses four key points that include patient selection, index test, reference standard, and flow and timing. According to the Standards for Reporting of Diagnostic Accuracy (STARD), the reference standard is considered to be the best available method for establishing the presence or absence of the condition of interest [28]. Various signaling questions, risk of bias and applicability concerns were judged as “low,” “high,” or “unknown”. Summary of QUADAS plot was generated by Review Manager Software (version 5.3.3, The Cochrane Collaboration).

### Statistical analysis

The pooled sensitivity, specificity, positive likelihood ratio [PLR, calculated as sensitivity / (1-specificity)], negative likelihood ratio [NLR, calculated as (1-sensitivity) / specificity], diagnostic odds ratio (DOR) and corresponding 95% confidence intervals (95% CI) were calculated from the TP, FP, FN, and TN values. DOR value is calculated as PLR/NLR [29]. The higher the value of DOR, the higher the diagnostic performance [30]. SROC and AUC were also generated. The effect of threshold was determined through the Spearman correlation between the logit of sensitivity and logit of 1-specificity. Cochran’s Q test was used to assess the heterogeneity caused by the non-threshold effect. The *P* value ≤0.05 and an inconsistency index (*I*^2^) value ≥50% indicated significant heterogeneity.

Sub-group analyses of SNVs were performed for genes (*PIK3CA*, *TP53*, and *ESR1*) and stages including early breast cancer (EBC including stages I-III) and advanced breast cancer (ABC including high risk stages III and IV). According to the NCCN guidelines, BC of stage III is referred to as locally advanced breast cancer (LABC). According to the ESO-ESMO 2nd international consensus guidelines, ABC comprises both LABC and metastatic breast cancer (MBC) [31]. However, in a study by Beaver (2014), stage III BC was classified as EBC [32]. In another study [33], patients diagnosed with BC at stages I-III were grouped together. Therefore, we grouped these studies into EBC subgroup [32, 33]. All the other studies with patients classified into MBC or ABC were grouped into the ABC subgroup.

A sensitivity analysis was also performed to explore the source of heterogeneity and the stability of pooled results. Deek’s funnel plot was generated to show the publication bias and the *p* value < 0.05 indicated the existence of a publication bias [34]. All the statistical analyses were performed using STATA software (version 12.0; STATA Corporation, College Station, TX) with the MIDAS module.

## Results

### Characteristics of identified studies

**Fig. 1:**
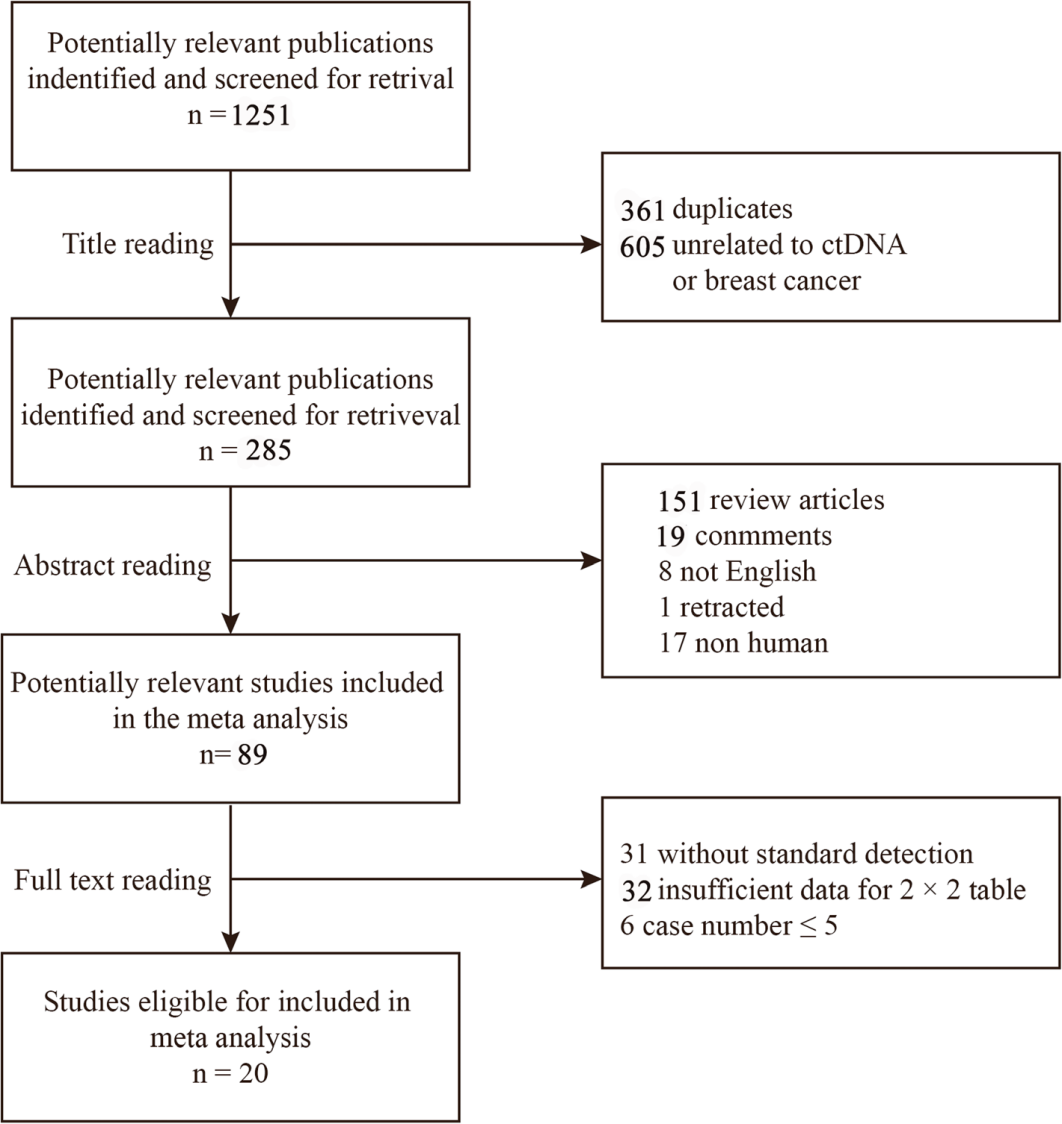
Flow diagram of study selection

Primary computerized literature search was used to identify 1251 records. However, after screening of the titles and abstracts, 1162 studies were excluded because they were either duplicate, non-English, review articles, non-human studies, retracted studies, comments, or irrelevant to the current study. Eighty-nine articles were further reviewed in detail. Out of these, 69 studies were further excluded because of insufficient data for making a 2 × 2 table or lack of standard detection. In a study by Garcia-Saenz JA [35], H1047R and E545K mutations in *PIK3CA* gene were detected separately. Because there is no study identifying the combination of H1047R and E545K mutations, these two data were included in this meta-analysis as independent studies. In the study by Beaver [32], although the patients came from the same population, the detection of cfDNA was conducted at “post-surgery” and “baseline”, respectively. Therefore, both studies were included. Finally, 20 studies including 1055 cases were identified as eligible (Fig. 1) for inclusion in the meta-analysis [8, 10–12, 32, 33, 35–48].

**Fig. 2:**
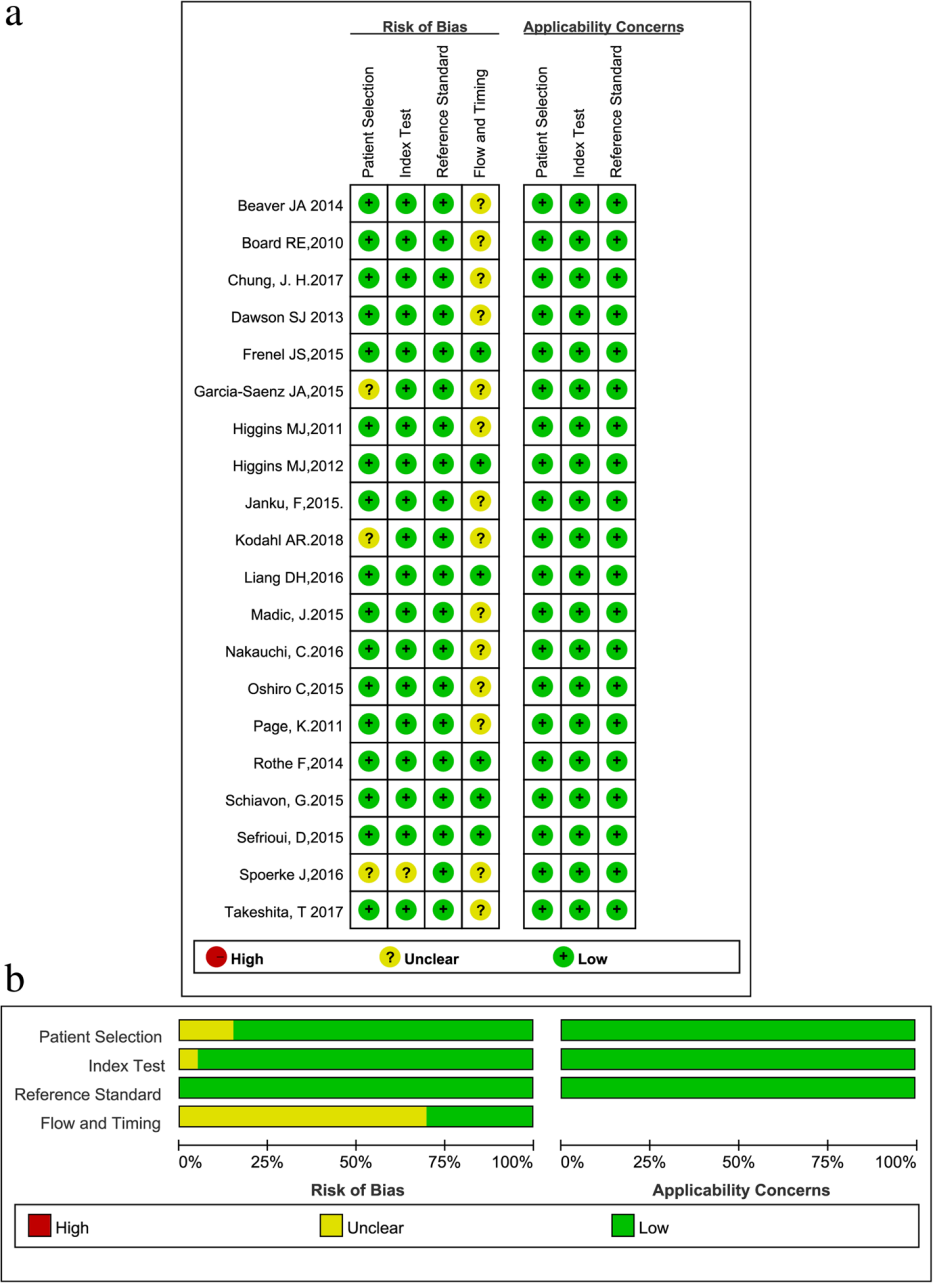
Methodological quality of eligible studies: Assessment of risk of bias based on the evaluation domains listed by each study (**a**) and presented as percentage across the included studies (**b**)

All eligible studies were published between 2010 and 2018. The QUADAS-2 summary plot is presented in Fig. 2. The main features of the eligible studies are summarized in Table 1.

**Table 1:** Characteristics of Eligible Studies

Author	Year	Case	Age (range)	Stage	Detection Method		Data sources	Sampling time	Gene	Mutation Type	TP	FP	FN	TN
					gDNA (tissue)	cfDNA (plasma)								
Beaver JA	2014	29	60 (38–77)	EBC	dPCR	dPCR	Reported in text	baseline	*PIK3CA*	SNV	13	0	1	15
Beaver JA (2)	2014	29	60 (38–77)	EBC	dPCR	dPCR	Reported in text	post-surgery	*PIK3CA*	SNV	10	3	0	16
Dawson SJ	2013	30	66 (43–85)	MBC	TAm-Seq/PE-WGS	dPCR,TAm-Seq	Reported in text	mid-therapy	*PIK3CA*	SNV	9	0	1	20
Dawson SJ(2)	2013	30	66 (43–85)	MBC	TAm-Seq/PE-WGS	dPCR,TAm-Seq	Reported in text	mid-therapy	*TP53*	SNV	15	0	1	14
Higgins MJ	2012	48	62(39–84)	MBC	BEAMing	BEAMing	Data-extrapolated	unavailable	*PIK3CA*	SNV	14	8	6	20
Rothe F	2014	17	48(35–62)	MBC	Ion PGM	Ion PGM	Reported in text	mid-therapy	*PIK3CA*	SNV	4	1	2	15
Rothe F (2)	2014	17	48(35–62)	MBC	Ion PGM	Ion PGM	Reported in text	chemotherapy	*TP53*	SNV	5	0	1	16
Spoerke J	2016	156	unavailable	MBC	dPCR	dPCR	Data-extrapolated	baseline	*PIK3CA*	SNV	54	8	15	79
Higgins MJ	2011	51	unavailable	MBC	sequencing	BEAMing	Data-extrapolated	unavailable	*PIK3CA*	SNV	14	12	0	25
Kodahl AR	2018	29	unavailable	MBC	dPCR	dPCR	Reported in text	unavailable	*PIK3CA*	SNV	20	0	4	5
Garcia-Saenz JA	2015	37	unavailable	ABC (IV 84%)	dPCR	dPCR	Data-extrapolated	unavailable	*PIK3CA* (p.E545K)	SNV	4	2	0	31
Garcia-Saenz JA (2)	2015	37	unavailable	ABC (IV 84%)	dPCR	dPCR	Data-extrapolated	unavailable	*PIK3CA* (p.H1047R)	SNV	6	2	4	25
Board RE	2010	30	64 (39–88)	MBC	ARMS	ARMS	Data-extrapolated	unavailable	*PIK3CA*	SNV	0	0	14	16
Board RE(2)	2010	43	59(43–79)	MBC	ARMS	ARMS	Data-extrapolated	mid-therapy	*PIK3CA*	SNV	8	1	2	32
Oshiro C	2015	313	≤50:121; > 50:192	EBC	real-time PCR	dPCR	Reported in text	preoperative	*PIK3CA*	SNV	85	0	25	203
Frenel,JS	2015	7	60 (29–78)	MBC	PGM	PGM	Reported in text	mid-therapy	*PIK3CA*	SNV	2	0	0	5
Frenel,JS (2)	2015	7	61 (29–78)	MBC	PGM	PGM	Reported in text	mid-therapy	*TP53*	SNV	4	0	2	1
Liang,DH	2016	23	55.5(55.5 ± 13.1)	ABC (IV/ high-risk III)	NGS	Digital Sequencing	Reported in text	mid-therapy	*PIK3CA*	SNV	4	1	2	16
Liang,DH (2)	2016	23	55.5(55.5 ± 13.1)	ABC (IV /high-risk III)	NGS	Digital Sequencing	Reported in text	mid-therapy	*TP53*	SNV	8	1	7	7
Schiavon G	2015	31	58(WT);69(MT)	ABC	dPCR	dPCR	Reported in text	relapsed or progressed	*ESR1*	SNV	3	0	1	27
Takeshita T	2017	35	56.4 (31–84)	MBC	dPCR	dPCR	Reported in text	mid-therapy	*ESR1*	SNV	1	4	5	25
Madic J	2015	31	unavailable	MBC	Hiseq and 454	Hiseq and 454	Reported in text	baseline	*TP53*	SNV	21	1	5	4
Nakauchi C	2016	17	57.7(32–80)	MBC	Ion-PGM	Ion-PGM	Reported in text	recurrent and primary	*PIK3CA*	SNV	3	2	1	11
Nakauchi C (2)	2016	17	57.7(32–80)	MBC	Ion-PGM	Ion-PGM	Reported in text	recurrent or primary	*TP53*	SNV	4	1	2	10
Sefrioui D	2015	7	55(41–71)	MBC	dPCR	dPCR	Reported in text	mid-therapy	*ESR1*	SNV	4	0	3	14
Janku F	2015	107	58 (20–84)	ABC	PBDS, MPD, Ion Torrent	BEAMing	Reported in text	mid-therapy	*PIK3CA*	SNV	12	8	2	85
Chung JH	2017	14	58 (32–85)	ABC (IV 94%)	HiSeq 2500/ 4000	HiSeq 2500 or 4000	Reported in text	mid-therapy	*PIK3CA*	SNV	3	1	0	11
Chung JH(2)	2017	14	58 (32–85)	ABC (IV 94%)	HiSeq 2500/ 4000	HiSeq 2500 or 4000	Reported in text	mid-therapy	*TP53*	SNV	4	2	0	8
Chung JH(3)	2017	14	58 (32–85)	ABC (IV 94%)	HiSeq 2500/4000	HiSeq 2500 or 4000	Reported in text	mid-therapy	*ESR1*	SNV	4	3	1	9
Chung JH(4)	2017	14	58 (32–85)	ABC (IV 94%)	HiSeq 2500/ 4000	HiSeq 2500 or 4000	Reported in text	mid-therapy	*CCND1*	CNV	1	0	4	9
Chung JH(5)	2017	14	58 (32–85)	ABC (IV 94%)	HiSeq 2500/4000	HiSeq 2500 or 4000	Reported in text	mid-therapy	*MYCN*	CNV	1	0	0	13
Liang DH(3)	2016	23	55.5(55.5 ± 13.1)	ABC (IV/high -risk III)	NGS	Digital Sequencing	Reported in text	mid-therapy	*ERBB2*	CNV	2	0	1	20
Liang DH(4)	2016	23	55.5(55.5 ± 13.2)	ABC (IV/high -risk III)	NGS	Digital Sequencing	Reported in text	mid-therapy	*EGFR*	CNV	1	2	1	19
Page K.	2011	30	unavailable	MBC	Quantitative PCR	Quantitative PCR	Reported in text	baseline	*HER2*	CNV	5	0	8	5

Abbreviation: *gDNA* genomic DNA, *cfDNA* cell free DNA, *EBC* early breast cancer, *dPCR* digital PCR, *FFPE* Formalin-fixed paraffin-embedded, *MBC* metastatic breast cancer, *TAm-Seq* tagged-amplicon deep sequencing, *PE-WGS* paired-end whole-genome sequencing, *BEAMing* beads, emulsion, amplification, magnetics, *ARMS* Amplification Refractory Mutation Testing System, *WT* wild type, *MT* mutation type, *ABC* advanced breast cancer, *NGS* Next generation sequence, *PBDS* PCR-based DNA sequencing

### Threshold effect and heterogeneity

For detection of SNVs, as shown in the Table 2 and Fig. 4, significant heterogeneity was noticed in the data accuracy, sensitivity, and specificity when all the studies were pooled. As for the EBC subgroup, the threshold effect analysis demonstrated that the Spearman correlation coefficient and *p* value were 1.00 and 0.00 (< 0.05) respectively, which suggests there is significant threshold effect among the studies of the EBC subgroup and it is not suitable to pool the effect-quantity of studies. On the other hand, for the ABC subgroup, the heterogeneity was reduced significantly. The Spearman correlation coefficient and p value were 0.02 and 0.92 (> 0.05) respectively, which suggests that there is no significant threshold effect among the ABC subgroup studies and the heterogeneity was not caused by threshold. Sensitivity analysis by single-study omission analysis for ABC subgroup revealed that the pooled results were significantly affected by the studies from Higgins (2011 and 2012) (Table 3). When these two studies were excluded, the heterogeneity was decreased significantly (*I*^2^ = 28.6%, *p* = 0.10 and *I*^2^ = 2.81%, *p* = 0.42). This shows that these two studies contributed to the high level of heterogeneity observed.

**Table 2:** Meta-analysis Estimates

SNV(Stage)	P Sen (95% CI)	Heterogeneity (I^2^, *p* value)	P Spe (95% CI)	Heterogeneity(I^2^,p value)	PLR (95% CI)	NLR (95% CI)	DOR	AUC (SROC)	ADT (SCC, p)
Overall	0.79(0.69–0.87)	50.0%, 0.00	0.94(0.90–0.97)	67.8%, 0.000	13.8(7.8–24.5)	0.22(0.15–0.34)	62(29–133)	0.95(0.92–0.96)	0.08, 0.5
EBC	0.79(0.04–1.00)	92.99%, 0.00	1.00(0.47–1.00)	93.30%, 0.000	1104.9(1.3–958,356.8)	0.21(0.01–7.30)	5174(22–1,242,093)	1.00(0.99–1.00)	1.00, 0.00
ABC	0.78(0.71–0.84)	35.72%, 0.04	0.92(0.87–0.95)	55.64%, 0.001	10.3(6.6–16.2)	0.24(0.18–0.32)	40(21–75)	0.91(0.88–0.93)	0.02, 092
ABC*	0.77(0.70–0.83)	28.6%, 0.10	0.93(0.90–0.95)	2.81%, 0.42	10.5(7.3–15.0)	0.25(0.18–0.34)	42(24–75)	0.94(0.92–0.96)	−0.09, 0.69
SNV(Gene)									
*PIK3CA*	0.83(0.68–0.91)	29.9%,0.12	0.95(0.90–0.98)	78.2%,0.000	15.5(7.6–31.5)	0.18(0.10–0.36)	84(33–219)	0.96(0.94–0.98)	0.23, 0.37
*PIK3CA*(EBC)	0.79(0.04–1.00)	92.99%, 0.00	1.00(0.47–1.00)	93.30%, 0.000	1104.9(1.3–958,356.8)	0.21(0.01–7.30)	5174(22–1,242,093)	1.00(0.99–1.00)	1.00, 0.00
*PIK3CA*(ABC)	0.80(0.74–0.85)	0.00%,0.45	0.91(0.86–0.96)	65.18%,0.00	9.0(5.3–15.5)	0.22(0.17–0.29)	41(21–80)	0.83(0.79–0.86)	0.35, 0.25
*PIK3CA*(ABC)^a^	0.80(0.73–0.85)	0.00%,0.78	0.93(0.90–0.95)	0.00%,0.91	11.1(7.6–16.1)	0.22(0.16–0.30)	50(29–88)	0.94(0.91–0.96)	0.10, 0.75
*TP53*	0.78(0.64–0.88)	39.72%,0.13	0.92(0.81–0.97)	3.56%,0.40	10.3(3.9–27.8)	0.24(0.13–0.42)	44(11–169)	0.94(0.91–0.95)	0.09, 0.87
*ESR1*	0.56(0.30–0.79)	45.67%, 0.14	0.95(0.69–0.99)	72.84%, 0.01	10.8(1.3–89.7)	0.47(0.25–0.88)	23(2–282)	0.80(0.76–0.83)	−0.05, 0.94
CNV	P Sen (95% CI)	Heterogeneity (I^2^,p value)	P Spe (95% CI)	Heterogeneity(I^2^,p value)	PLR (95% CI)	NLR (95% CI)	DOR	AUC (SROC)	ADT (SCC, p)
	0.42 (0.24–0.62)	0.0%,0.52	0.98(0.71–1.00)	13.27%, 0.33	19.9(1.1–365.1)	0.60(0.42–0.84)	33(2–702)	0.45(0.41–0.50)	−0.50, 0.39

Note: a, Studies of Higgins MJ.2011 and Higgins MJ. 2012 were excluded

Abbreviation: *P Sen* Pooled Sensitivity, *CI* confidence interval, *P Spe* Pooled Specificity, *PLR* Positive Likelihood Ratio, *NLR* Negative Likelihood Ratio, *DOR* Diagnostic Odds Ratio, *AUC* Area Under Curve, *ADT* Analysis of Diagnostic Threshold, *SCC* Spearman correlation coefficient, *EBC* Early breast cancer, *ABC* Advanced breast cancer

**Table 3:** Sensitivity Analysis

SNV (ABC)	Author(Study)	Year	Sensitivity	Heterogeneity (*I*^2^, p value)	Specificity	Heterogeneity (*I*^2^, p value)
	Board RE.2010(2)	2010	0.774(0.715–0.818)	40.6%, 0.021	0.894(0.871–0.923)	53.1%, 0.001
	Chung.JH.2017	2017	0.772(0.720–0.819)	38.3%, 0.031	0.898(0.876–0.922)	55.4%, 0.001
	Chung.JH.2017(2)	2017	0.771(0.719–0.818)	36.4%, 0.035	0.905(0.878–0.927)	54.9%, 0.001
	Chung.JH.2017(3)	2017	0.774(0.716–0.818)	40.6%, 0.024	0.906(0.880–0.929)	53.4%, 0.000
	Dawson SJ.2013	2013	0.770(0.717–0.817)	38.9%, 0.028	0.895(0.867–0.919)	51.3%, 0.002
	Dawson SJ.2013(2)	2013	0.760(0.704–0.810)	34.0%, 0.054	0.896(0.88–0.920)	52.6%, 0.001
	Frenel JS.2015	2015	0.773(0.721–0.818)	39.8%, 0.024	0.898(0.870–0.922)	54.2%, 0.001
	Frenel JS.2015(2)	2015	0.777(0.724–0.823)	40.1%, 0.031	0.899(0.871–0.922)	55.2%, 0.000
	Garcia-Saenz JA.2015	2015	0.773(0.719–0.821)	39.4%, 0.026	0.896(0.876–0.926)	59.5%, 0.000
	Garcia-Saenz JA.2015(2)	2015	0.780(0.728–0.827)	36.4%, 0.040	0.897(0.869–0.922)	55.2%, 0.000
	Higgins MJ.2011	2011	0.777(0.722–0.825)	37.4%, 0.034	0.902(0.875–0.925)	59.5%, 0.000
	Higgins MJ.2012	2012	0.763(0.709–0.808)	25.2%, 0.129	0.914(0.892–0.939)	43.9%, 0.012
	Janku. F.2015	2015	0.780(0.726–0.825)	39.0%, 0.027	0.912(0.886–0.934)	42.7%, 0.001
	Liang DH.2016	2016	0.770(0.717–0.815)	38.8%, 0.028	0.896(0.865–0.926)	55.1%, 0.000
	Liang DH.2016(2)	2016	0.777(0.719–0.821)	39.4%, 0.026	0.897(0.875–0.925)	55.4%, 0.000
	Madic.J.2015	2015	0.787(0.729–0.831)	32.3%, 0.066	0.899(0.871–0.926)	55.4%, 0.000
	Nakauchi.C.2016	2016	0.771(0.717–0.820)	39.5%, 0.025	0.900(0.872–0.923)	55.0%, 0.001
	Nakauchi.C.2016(2)	2016	0.775(0.717–0.819)	39.9%, 0.024	0.904(0.877–0.927)	55.1%, 0.000
	Rothe F.2014	2014	0.777(0.719–0.821)	39.4%, 0.026	0.899(0.871–0.926)	55.4%, 0.001
	Rothe F.2014(2)	2014	0.777(0.719–0.821)	39.4%, 0.026	0.898(0.875–0.925)	55.1%, 0.001
	Schiavon.G.2015	2015	0.773(0.721–0.820)	40.5%, 0.022	0.896(0.867–0.920)	52.2%, 0.000
	Sefrioui.D.2015	2015	0.775(0.723–0.821)	40.7%, 0.021	0.894(0.85–0.918)	49.6%, 0.003
	Spoerke J.2016	2016	0.779(0.727–0.826)	38.6%, 0.034	0.896(0.874–0.924)	52.6%, 0.001
	Takeshita.T.2017	2017	0.787(0.712–0.825)	19.3%, 0.021	0.901(0.867–0.927)	55.3%, 0.001
	Kodahl AR.2018	2018	0.769(0.715–0.818)	39.8%, 0.024	0.898(0.878–0.928)	54.4%, 0.001
CNV	Author(Study)	Year	Sensitivity	Heterogeneity (I^2^, p value)	Specificity	Heterogeneity (I^2^, p value)
	Chung.JH.2017(4)	2017	0.474(0.244–0.711)	0.00%, 0.499	0.966(0.883–0.996)	29.6%, 0.235
	Chung.JH.2017(5)	2017	0.391(0.197–0.615)	0.00%, 0.600	0.964(0.875–0.996)	24.5%, 0.264
	Liang DH.2016(3)	2016	0.381(0.181–0.616)	0.00%, 0.422	0.958(0.857–0.995)	12.3%, 0.331
	Liang DH.2016(4)	2016	0.409(0.207–0.636)	17.1%, 0.305	1.000(0.925–1.000)	0.00%, 1.000
	Page.K.2011	2011	0.455(0.167–0.766)	15.8%, 0.210	0.968(0.890–0.996)	33.7%, 0.210

For CNV, the heterogeneity of sensitivity and specificity were 0.0% (*p* = 0.52) and 13.27% (*p* = 0.33), respectively. The Spearman correlation coefficient and *p* value were − 0.50 and 0.39 (> 0.05), respectively, which suggests there is no significant heterogeneity and threshold effect among the studies involving detection of CNVs.

### Publication bias

**Fig. 3:**
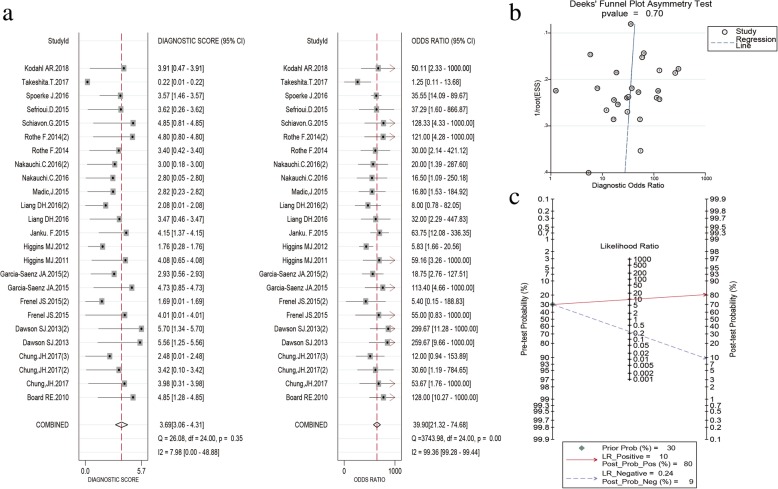
Diagnostic odds ratio (DOR), Deek’s funnel plot and Fagan’s Nomogram of cfDNA for SNV (ABC): **a**. The DOR of SNV was 40 (21–75) indicating high diagnostic performance; **b**. The *p* value was 0.70 (> 0.05) which suggests that there was no evidence of publication bias for SNV. **c**. The post-test probability of positive result was raised from 30 to 80%, which indicates a high diagnosis utility of cfDNA for detection of SNV

For SNV, the publication bias tested using the Deek’s funnel plot was 0.70 (> 0.05) (Fig. 3b). This suggests that there is no evidence of publication bias for SNV studies. Since CNV detection studies are less than 10, it is not suitable to perform this analysis on CNV studies.

### Diagnostic accuracy

**Fig. 4:**
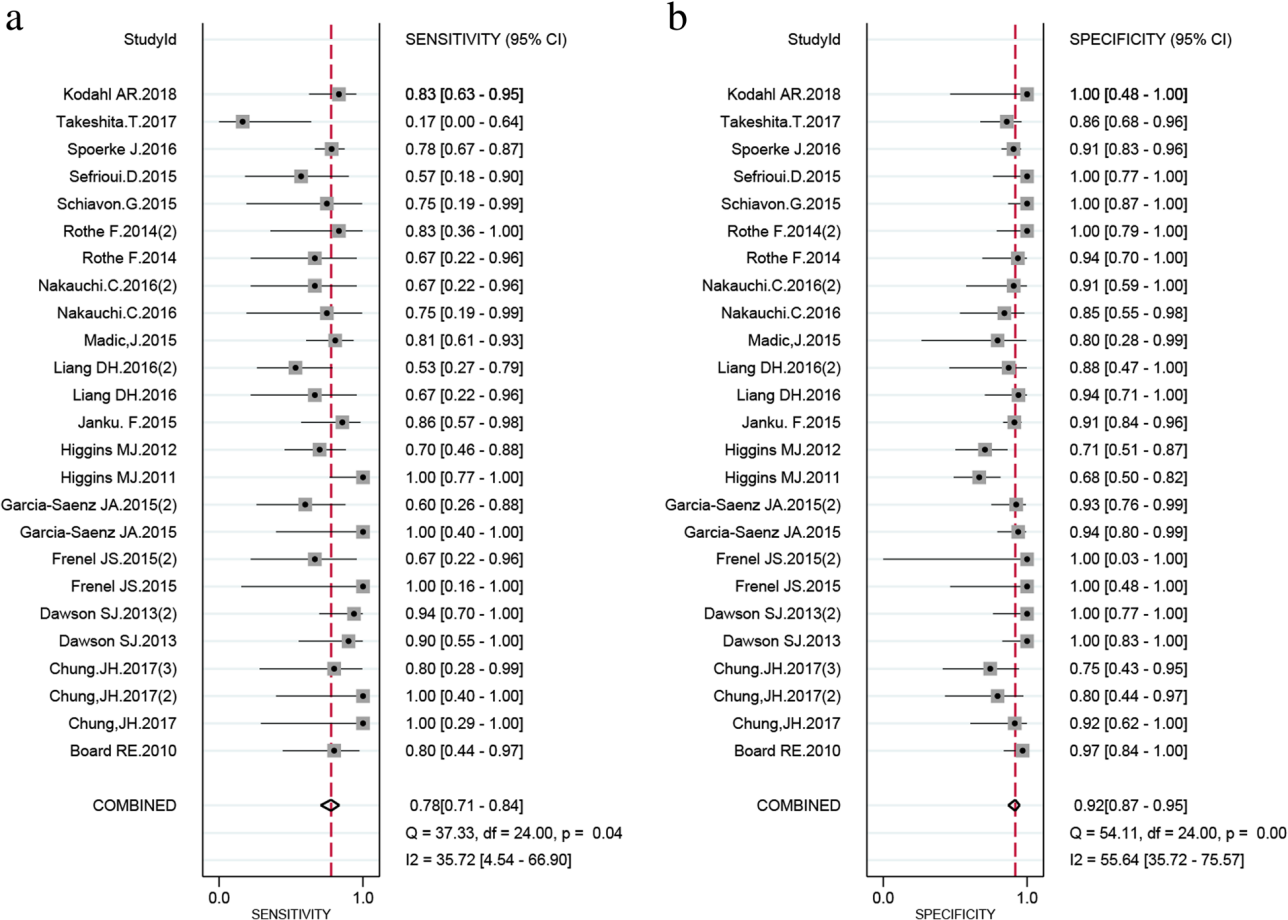
The pooled sensitivity (**a**) and specificity (**b**) of cfDNA for SNV (ABC) detection were 0.78 (0.71–0.84) and 0.92 (0.87–0.95), respectively which suggests that cfDNA has a higher specificity for SNV detection

**Fig. 5:**
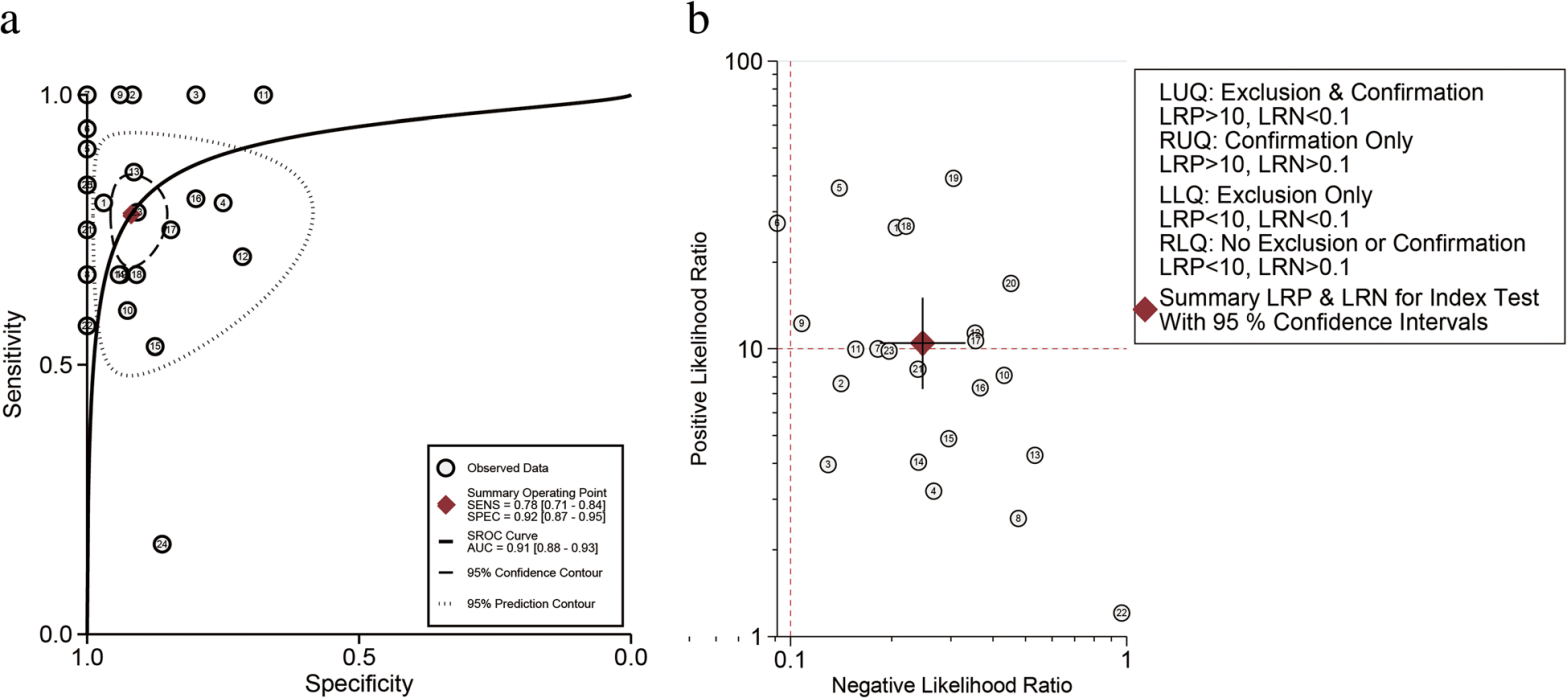
Summary roc curve (SROC) and Summary PLR and NLR for cfDNA test: **a**. SROC of cfDNA based detection of SNV (ABC): The area under the curve (AUC) was 0.91 (0.88–0.93) indicating an impressive overall accuracy; **b**. Summary PLR and NLR for cfDNA based detection of SNV (ABC): The PLR and NLR were 10.3 (6.6–16.2) and 0.24 (0.18–0.32), respectively, indicating that the detection of SNV through cfDNA has a highly significant detection rate but exhibits a poor exclusion ability

For SNV (ABC), compared to the reference standard test, the pooled sensitivity and specificity were 0.78 (0.71–0.84) and 0.92 (0.87–0.95), respectively. The PLR, NLR and DOR were 10.3 (6.3–17.2), 0.24 (0.18–0.33), and 40 (21–75), respectively. The SROC exhibited an AUC of 0.91 (0.88–0.93) (Table 2 and Figs. 3a, 4, and 5a). After the studies by Higgins (2011 and 2012), which contributed mainly to the heterogeneity were excluded, the results of these indicators changed very slightly (Table 2). The pooled results of different genes subgroups are shown in Table 2. The diagnostic performance of different genes was different, such as AUC, *PIK3CA* and *TP53* exhibited the values of 0.96 (0.94–0.98), 0.94 (0.91–0.96) respectively, while *ESR1* showed the lowest value 0.80 (0.76–0.83).

**Fig. 6:**
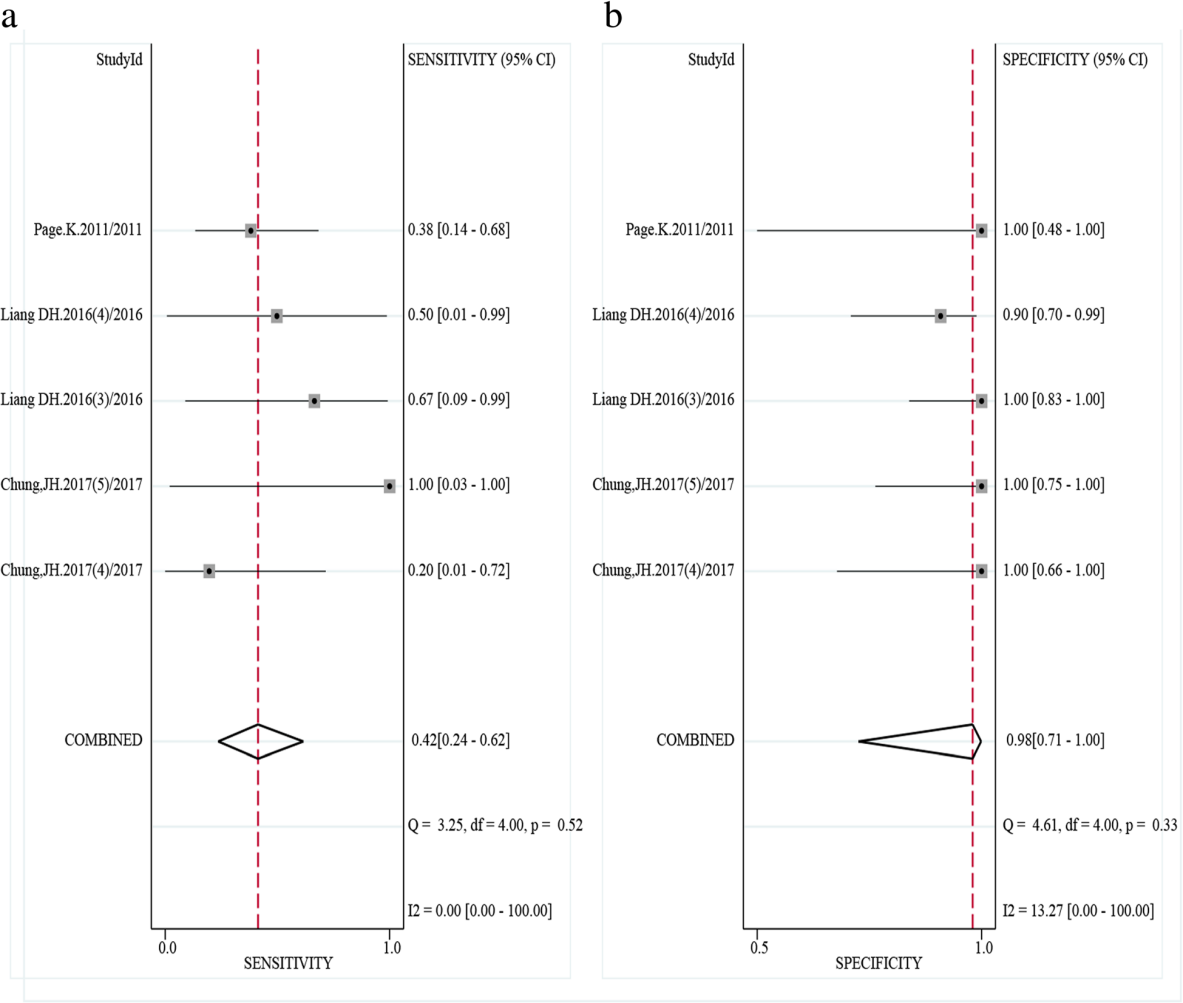
The pooled sensitivity (**a**) and specificity (**b**) of cfDNA for CNV detection were 0.42 (0.24–0.62) and 0.98 (0.71–1.00), respectively

For CNV, the pooled sensitivity, specificity, PLR, NLR, DOR and AUC were 0.42 (0.24–0.62), 0.98 (0.71–1.00), 19.9 (1.1–365.1), 0.60 (0.42–0.84), 33 (2–702) and 0.45 (0.41–0.50) respectively (Table 2 and Fig. 6).

## Discussion

This study is the first study involving the evaluation of the diagnostic accuracy of cfDNA for detection of different mutation types (SNV and CNV) and for different genes. Currently, there are other meta-analysis studies on the diagnostic values of cfDNA in BC, such as studies from Wang H et al. and Lin Z et al. [49, 50]. But these studies focus on the quantitative or qualitative evaluation of cfDNA for the diagnosis of BC and the identification of benign breast disease. The results of these studies suggest that plasma cfDNA is of great importance in the screening and diagnosis of breast cancer. However, the current study was mainly designed to evaluate the consistency of non-invasive cfDNA detection of gene mutations using tissue DNA detection as a standard reference.

For SNV (ABC), analysis results of ABC subgroup show that mutation detection has a high degree of consistency between cfDNA and biopsy tissue DNA. Although the pooled results including sensitivity, specificity, PLR, DOR and AUC (0.78, 0.92, 10.3, 40 and 0.91) were all lower than the previous study (0.91, 0.98, 39.0, 428 and 0.99) [24], because the present study included more reports and more genes (*PIK3CA*, *TP53*, and *ESR1*), the conclusions drawn are theoretically more reliable.

Fagan’s plot was generated for the visual presentation of the clinical utility of cfDNA. The results revealed that the post-test probability of positive result was raised from 30 to 80% (Fig. 3c). PLR > 10.0 and NLR < 0.1 was defined generally as clinically useful test. In this study, the pooled PLR and NLR of SNV (ABC) reached 10.3 and 0.24, respectively, indicating that the detection of SNV through cfDNA has significantly high detection rate but exhibits a very low ability for exclusion (Fig. 5b, Table 2). In other words, SNV detection using cfDNA qualified as a confirmative assay although it may not be suitable to be used as a test for exclusion. There are also differences among the several common genes, and according to AUC, the diagnostic value of cfDNA for *PIK3CA* and *TP53* is higher than *ESR1*. This study suggests that for the patients with ABC, the detection of genetic mutations by cfDNA has a high utility of being used as a surrogate of tissue DNA, yet reliable results cannot be obtained in EBC patients because of the obvious heterogeneity.

In the case of CNV, the meta-analysis results showed a good homogeneity among the studies evaluating the use of cfDNA for the detection of CNV. Owing to low sensitivity and AUC compared with the tissue DNA based detection (Table 2), cfDNA is not very suitable for the detection of CNVs. The reliable conclusions depend on more published research results which can be included in our study. However, as the primitive attempt to Meta-analyze the diagnostic value of cfDNA for detection of CNVs, it still has important significance which can attract more interested researchers to conduct further study.

False negatives observed for cfDNA mainly because of the cfDNA detection limits such as the recovery of cfDNA or non-biological errors deriving from library preparation and sequencing, represent a main barrier for employing super-sensitive cfDNA for identification of markers [51]. But this barrier can be overcome by plasma DNA extraction and new high efficiency methods for enrichment and capture in sequencing. Thus, the analytical sensitivity and specificity can be further improved.

In addition, there are some factors that may cause differences in tissue DNA and cfDNA test results, resulting in the heterogeneity between studies and a bias in the final results. For example, the time of tissue collection or surgery and/or administration of systemic therapy relative to the blood collection, differences owing to the use of stored and fresh biological specimens, differences in the detection methods used for tissue and blood in some studies, and variability of the cfDNA detection methods used. Therefore, in order to get more reliable results, more rigorous inclusion criteria should be set, and tissue and blood samples should be obtained at the same time point. More detailed subgroup design may be required, such as the before treatment, after treatment, different treatment method, different specimen storage time, and different detection method subgroups.

However, there are some limitations of this meta-analysis. Firstly, several studies were small scale, which might lead to a bias. The Deek’s funnel plot showed that there is no evidence of publication bias for SNV. But there are very few studies on CNV to test for the publication bias. Thus, more reliable results require more research reports for CNV detection using cfDNA. Secondly, significant heterogeneity was observed in the SNV detection studies. We explored the source of heterogeneity by subgroup analysis, threshold effect analysis and single-study omission analysis. Because of significant heterogeneity in EBC subgroup, these studies were not pooled into meta-analysis. For the studies of ABC subgroup, after studies of Higgins (2011 and 2012) were omitted, high level of detection accuracy was observed as shown in the Table 2, indicating that these two studies may be the primary source of heterogeneity. Thirdly, only studies in English were included in this meta-analysis, but there are still several studies written in non-English language that must be taken into consideration. Fourthly, only the studies on the gene mutation analysis using cfDNA in BC were included. There is a more sensitive method for detection of mutation in cfDNA such as integrated digital error suppression (iDES) [51]. But this study was about other cancers instead of BC so it was excluded for this meta-analysis. This may lead to the under-representation of the performance of cfDNA based mutation detection. Fifthly, owing to the significant heterogeneity, the results from EBC subgroup could not be included in the pooled analysis. More homogeneous studies are needed to evaluate the combined diagnostic value of detection of gene mutations by cfDNA. Sixthly, molecular classification of tumors is of great significance for predicting the risk of recurrence and metastasis of breast cancer and its response to treatment. BC is currently classified into four intrinsic subtypes: Luminal A, Luminal B, ‘basal-like,’ and Erb-B2 overexpression subtype [52]. But in the studies included in this meta-analysis, there is no sufficient data presented for describing or calculating sensitivity and specificity values based on the molecular classification. Therefore, in this meta-analysis study, we did not perform subgroup analyses by molecular classification.

## Conclusions

In conclusion, this meta-analysis shows that SNV detection through cfDNA has a high sensitivity, specificity, and accuracy, when the detection with DNA isolated from tissue samples was used as the standard reference. Therefore, it is a promising alternative tool to the tumor tissue for detection of SNV in BC. But for CNV, there is a need for further exploration.

## Abbreviations

ABC: Advanced breast cancer
AUC: Area under the curve
BC: Breast cancer
cfDNA: Cell-free tumor DNA
CNV: Copy number variant
DOR: Diagnostic odds ratio
EBC: Early breast cancer
FN: False negative
FP: False positive
NLR: Negative likelihood ratio
PLR: Positive likelihood ratio
SNV: Single nucleotide variant
SROC: Summary receiver operative curve
TN: True negative
TP: True positive
